# Effect of pecuniary costs and time costs on choice of healthcare providers among caregivers of febrile children in rural Papua New Guinea

**DOI:** 10.1186/s13561-019-0250-x

**Published:** 2019-12-11

**Authors:** Takahiro Tsukahara, Takuma Sugahara, Seiritsu Ogura, Francis Wanak Hombhanje

**Affiliations:** 1 Department of International Affairs and Tropical Medicine, Tokyo Women’s Medical University, 8-1 Kawada-cho, Shinjuku-ku, Tokyo, 162-8666 Japan; 2 School of Economics, Hosei University Graduate School, 2-15-2 Ichigaya Tamachi, Shinjuku-ku, Tokyo, 162-0843 Japan; 3 Centre for Health Research and Diagnostics, Divine Word University-Rabaul Campus, Kokopo, Papua New Guinea

**Keywords:** Health demand, Community health worker, Time costs, Choice set formation, Mixed logit, Oceania, Pacific

## Abstract

**Background:**

User fees, transportation costs, and time costs impair access to healthcare by rural communities in low and middle income countries. However, effects of time costs on demand for healthcare are less understood than effects of user fees for health providers. In addition, prospective patients might not know about all health services available. This study aims to investigate how the family caregivers of febrile children respond to the pecuniary costs and time costs in their choice of health providers in rural Papua New Guinea.

**Methods:**

Using an original questionnaire, we surveyed households in the catchment area surrounding Dagua Health Center in East Sepik Province, Papua New Guinea, during February–March 2015. We estimated the probability of choosing one among four categories of providers (i.e., the health center, aid posts, village health volunteers [VHVs], or home-treatment) via a mixed logit model in which we restrict alternatives to those for which family caregivers knew cost information.

**Results:**

Of 1173 family caregivers, 96% sought treatment for febrile children from four categories of providers. Almost all knew the location of the health center and a health volunteer, but only 50% knew the location of aid posts. Analysis by discrete choice model showed that pecuniary costs and time costs were inversely associated with the probability of choosing any type of provider. We then changed pecuniary costs and time costs counterfactually to calculate and compare the probability of choosing each provider. Time costs affected the choice more than pecuniary costs, and individual heterogeneity appeared among caregivers with respect to pecuniary costs. When pecuniary or time costs of VHVs are altered, substitution between VHVs and home-treatment appeared.

**Conclusions:**

Our findings suggest that policies to increase awareness of aid posts and reduce time costs in addition to treatment fees for each category of healthcare provider could help developing economies to improve access to essential healthcare services.

## Background

Access to essential healthcare services is an important public health issue in low and middle income countries (LMIC). Though health policy causing change of supply curve (e.g., increasing quality and quantity of health staff and medial materials) has been promoted, the importance of intervention overcoming demand side barriers was also emphasized to improve healthcare service utilization [1]. Among demand side barriers, the previous economic studies has intensively argued the effect of user fees on healthcare access in LMIC [2–8]. Although quality of the evidence is poor, a review on intervention studies suggests that user fees have a negative effect on the use of medical services [9].

On the other hand, Acton [10] pointed out that non-monetary costs can be an important factor affecting the use of health services. In LMIC, Heller [2] initially analyzed health provider choice including non-monetary costs such as travel times and waiting times using the discrete choice model. In several pioneer works in LMIC, non-monetary costs as well as pecuniary costs were included into discrete choice models [2–5, 8, 11]. However, these studies mainly focused on the effect of user fee on healthcare service utilization in the context of the structural adjustment programs, and in most studies, amounts of non-monetary costs were relatively small because the target population lived in urban areas.

The population living in rural parts of LMIC countries, however, confront long distances, undependable transportation, and steep travel costs to access healthcare. Numerous studies examine how distance traveled affects choice of healthcare providers there [12–16]. In rural parts of LMIC, however, time costs of visiting providers can be quite large and hence should not be ignored. The studies that have left out travel time may have overstated the importance of distance or travel costs on provider choice. More distant patients may visit the provider less often, not only because it costs more for them to visit, but also because it takes much longer for them to visit. Yet, in rural parts of LMIC, little is known about the effect of time cost on healthcare provider choice except in a few reports in maternal and child health field [17, 18]. Moreover, once we know more about the effects of time costs there, we may be able to find policies that can reduce time costs, and we may be able compare them with the policies that reduce monetary costs of patients.

In Papua New Guinea (PNG), as the mortality rate for children under 5 years old is the third worst among 23 developing East Asia and Pacific Region economies [19], policies to control the disease and acute infections in children has been given a high priority in public health; improvement in child mortality is a goal of PNG’s national health plan [20]. Therefore, we analyze which type of healthcare provider the residents in rural PNG choose to take their children with fevers, a symptom of acute infection for treatment. Although fees for formal healthcare services are fixed for an episode and relatively inexpensive, residents face differential costs for travel, time, and treatment options [16]. Price-substitutability among alternatives is easily estimated, allowing us to compare how pecuniary and time costs affect the probability of choosing each type of provider.

This study examines how the family caregivers of febrile children respond to the pecuniary costs and time costs in their choice of health providers. We used discrete choice models to estimate the probability of an alternative being chosen. We calculated own- and cross-cost responsiveness to predicted choice probability for each provider by counterfactual analysis and examined substitutability between alternatives. We also performed a sub-group analysis for severity of minor patients’ symptoms.

## Methods

### Study setting

The study covered 23 villages in the catchment area surrounding the Dagua Health Center, located 56 km west of Wewak, the capital of East Sepik Province of PNG. In the lowland swamp along the main coastal road malaria is endemic year-round. The Dagua Health Center is operated 24 h a day by the Catholic Church. Eleven health professionals provide medical/public health services, including ambulatory care, hospitalization for general internal medicine (tuberculosis, antenatal and normal delivery care), and outreach services (immunization and emergency care). It treated an average of 20.1 outpatients per day in 2016, a figure not considered to constitute excessive demand [21].

With only one health professional assigned, each aid post provides general outpatient care and normal delivery services. Its staff is nationally certified by completing a two-year program at a medical college or a university and is allowed to perform the same clinical works as nurses. Our surveyed population had access to four such aid posts. Although records on the number of their patients were unavailable, we observed that two or fewer patients awaited treatment at any given time, suggesting the demand for their services was not excessive. While the traditional practitioners of herbal or spiritual remedies were commonplace, the area had only one distributor of Western pharmaceuticals. The provincial capital city of Wewak, has only one general hospital and two public clinics. The hospital provides general medicine, surgery, pediatric, obstetric, psychiatric, and physical therapy in emergency outpatient and inpatient departments besides overseeing provincial public health. Staffed by five or so professionals, public clinics provide general outpatient and normal delivery services.

Outpatient fees at these formal healthcare facilities are capitation payments, covering consultation, clinical exams, laboratory tests, medications, and follow-up visits. The fees vary from one facility to another, but the costs are less than the nation’s minimum hourly wage. Public transportation, in the form of buses or public motor vehicles (PMVs), are available to villagers beyond walking distance. PMVs operate daily, except on Sundays, from villages to Wewak, once in the morning, and from Wewak to villages, once in the afternoon. The government regulates transportation charges on the basis of distance traveled. Neither public nor community-based health insurance had been introduced in the area studied.

To improve access to basic healthcare, lay workers called “village health volunteers” (VHVs) have served in area studied since 2007 [16]. The VHVs treat patients with acute infectious diseases such as malaria, pneumonia, and diarrhea without charge, but, with approval from local authorities, they may charge a small flat rate (less than fees of formal health facilities). During the period studied, a VHV treated an average of 0.81 patients per day, the one with the smallest load treating 0.25 patients per day and the one with the highest load treating 1.71 patients per day. We never observed VHVs refusing a consultation or curtailing services due to excessive patient load. Excess demand is unlikely.

### Study design and data collection

During February–March 2015 using an original questionnaire, we conducted a cross-sectional survey of households in which parents or parent surrogates were taking care of children under age 15. Trained field interviewers collected data about episodes of fever among their children and the respondent’s choices of health providers during the 2 weeks preceding the interview. The lead researcher double-checked questionnaires completed by field interviewers. Missing and erroneous values were corrected by revisiting respondents.

Questions asked about instances of fever, home-treatment at its onset, initial choices of health provider, the second, and the third or subsequent choices of health provider. The survey also asked the caregivers to give information on all health providers where they may take their children when they are sick: namely, [1] location, [2] name, [3] out-of-pocket payment, [4] wait/treatment time, [5] time spent from house to the provider when they travel only on foot, [6] respondent’s payment for a round-trip to the provider when they use public transportation, and [7] clock time of leaving home and clock time of returning home when they use public transportation. It also asked respondents about the characteristics and the conditions of the minor patients (gender, age, severity of illness as perceived by respondents), years of schooling of the caregivers, number of individuals in the household, holdings of western drugs for fever treatment (e. g., acetaminophen, amoxicillin, antimalarial drugs), information on household assets (ownership of mobile phones, radios, generators, cars or outboard motorboats, tin roofs, and brick, metal, or concrete walls), and information on the access to safe drinking water.

We define the pecuniary costs as “caregiver’s out-of-pocket payments for a health provider plus round-trip transportation fees to get there”. We use survey respondents’ self-reported estimate of pecuniary costs for each healthcare provider respondents had named, including the ones they had not chosen. As we described above, out-of-pocket payment for a health provider was equal to a fixed sum covering consultation, clinical exams, laboratory tests, treatments, drugs, and follow-up visits for VHVs, the aid posts, or the health center.

Time cost is defined as “the caregiver’s opportunity cost for his/her self-reported time spent in seeking and obtaining healthcare away from home or work”. To calculate time costs, first we calculate time required for care at each health provider for each caregiver. When caregivers would travel to a health provider only on foot, total time required for its care was calculated as round-trip walking time plus its wait/treatment time. When caregivers would use public transportation to a health provider, total time required for its care was calculated as time difference between clock time of returning home and clock time of leaving home. We then calculate the individual time costs by multiplying the time required for care at each health provider by the caregiver’s wage rate.

To check the self-reported travel time, we calculated individual travel distances for each provider. We first recorded the locations of each house and each health provider with global positioning system (GPS) devices (Foretrex 401, Garmin Ltd). We then measured individual distances, using a digital map of the area (PASCO Satellite Ortho, PASCO Corporation) and Quantum GIS 2.14, as [1] the walking distance for a round trip from home to the provider, if the caregiver who would travel on foot, and as [2] the sum of walking distance from house to road, the public transportation distance (i.e., actual road distance), and walking distance from road to the provider, if the caregiver would travel on foot and by public transportation.

### Statistical analysis

We calculated the probability for a caregiver to choose a particular type of healthcare provider using a discrete choice model in which the individual tries to maximize utility by their choice. However, our respondents were not necessarily aware of all the providers available in the area. Providers they were unaware of could not have been placed in the set of eligible alternatives. Also, conceivably, respondents would not choose providers they believe offer low utility even if they were aware of them. For these reasons, we postulated that to be an eligible alternative, [1] a health provider had to be known to the respondent, and [2] the probability of choosing it had to be at least 2% in our pooled data. We calculated pecuniary and time costs only for the alternatives meeting those two criteria.

The model’s explained variable was “a healthcare provider chosen for a febrile child in the two weeks before the interview date.” We defined home-treatment as all the treatment given within a household at any time during one fever episode without incurring any pecuniary costs. Home-treatment, for instance, included monitoring the sick child without treatment in a family. Following the previous studies, time costs of home-treatment were normalized to zero [4, 8].

When a caregiver did not have cost information of the health center, any aid post, or any VHV, we excluded those alternatives from his/her choice set. We assumed all caregivers could have chosen home-treatment. Therefore, the number of alternatives in a choice set varied for each caregiver between two to four, and its average was 3.4. A small number of respondents visited multiple healthcare providers for the same episode. In such cases, we set the first provider visited as the explained variable.

If alternative-specific variables (i.e., pecuniary costs and time costs) alone have a random component, utility for the choice of alternative *j* by respondent *i* is given as

1 $$ {U}_{ij}={x}_{ij}^{\prime }{\beta}_i+{z}_i^{\prime }{\gamma}_j+{\varepsilon}_{ij}={x}_{ij}^{\prime}\beta +{z}_i^{\prime }{\gamma}_j+{x}_{ij}^{\prime }{v}_i+{\varepsilon}_{ij}, $$

where *x*_*ij*_ is a vector of alternative-specific variables. *z*_*i*_ is a vector of respondent-specific variables. *ε*_*ij*_ is the error term, which mixed logit models assume follows an extreme value distribution. In Eq. (1), *β*_*i*_ = *β* + *v*_*i*_, where *v*_*i*_ denotes random coefficients. The logit probability of alternative *j* selected by respondent *i* is represented as

2 $$ {P}_{ij}\mid {v}_i=\frac{1(j)\times \exp \left({x}_{ij}^{\prime}\beta +{z}_i^{\prime }{\gamma}_j+{x}_{ij}^{\prime }{v}_i\right)}{\sum \limits_{l=1}^J1(j)\times \exp \left({x}_{il}^{\prime}\beta +{z}_i^{\prime }{\gamma}_l+{x}_{ij}^{\prime }{v}_i\right)},j=1,\dots, J, $$

where 1(*j*) takes a value of 1 if a respondent knows cost-related information of alternative *j* and 0 otherwise. Probability of choice is the integral of the logit probability over density function *v*_*i*_. Assuming *f*(*v*) is a probability density function of *v*_*i*_ with a normal distribution, the probability of choosing alternative j selected by respondent i is described as

3 $$ {P}_{ij}=\int \left(\frac{1(j)\times \exp \left({x}_{ij}^{\prime}\beta +{z}_i^{\prime }{\gamma}_j\right)}{\sum \limits_{l=1}^J1(j)\times \exp \left({x}_{ij}^{\prime}\beta +{z}_i^{\prime }{\gamma}_j\right)}\right)\ f(v) dv,j=1,\dots, J. $$

We used Stata15 (StataCorp, Texas, USA) and the command *asmixlogit*. Simulation methods with 500 Halton draws approximate the maximum log likelihood.

Individual-specific variables were the minor child’s gender, age, perceived severity of illness, caregiver’s education, presence of Western drugs in the household, household size (number of persons), and an index of household assets. Assets were selected to proxy long-term wealth by constructing a linear index of asset ownership and housing characteristics using principle component analysis [22]. Seven dummies estimate the index: owning a mobile phone, owning a radio or stereo, living a house with tin roof, living a house with a Western-style wall, owning a generator, having safe drinking water, and owning a car or outboard motorboat [16].

## Results

### Awareness of healthcare services and formation of choice sets

Our results reveal that almost all (98%) of caregivers had the information on the location of Dagua Health Center and VHVs, while 65% of them on the locations of public clinics in Wewak, 56% of them on traditional health practitioners, 54% of them on aid posts, and 12% of them on the dispenser of Western pharmaceuticals (Table 1). Caregivers indicated that 493 of the 2679 minors (or 18% of minors) living in the surveyed households had episodes of fever during the survey period. Treating the febrile minor at home was the most common choice (40%), followed by visiting VHVs (34%), the health center (13%), and the aid posts (9%). These four alternatives accounted for 96% (or 475) of 493 reported choices. Other healthcare providers included traditional practitioners (1.8%, or 9 responses), outreach by health center staff (0.6%, or 3 responses), public clinics in Wewak (0.4%, or 2 responses), dispensers of Western drugs (0.4%, or 2 responses), and unknown (0.4%, or 2 responses). No visits to Wewak General Hospital were reported. As noted above, we excluded alternatives with probabilities below 2% from the individual choice sets. Consequently, the choice of the four alternatives, namely, home-treatment, VHVs, the health center, and the aid posts remained for analysis as our explained variable.

**Table 1:** Awareness of health provider’s location

Health service	N	Awareness
Health center	1167	0.983
VHV	1168	0.979
Clinic	1165	0.658
Traditional health practitioner	1167	0.559
Aid post	1162	0.540
Western medicine seller	1156	0.143

Percentages of the survey’s 1173 respondents who are aware of healthcare providers’ locations are shown in the far right column

*VHV* village health volunteer

### Adjustment of time costs

Self-reported travel time correlated to the measured distance traveled (correlation coefficient: 0.755 for health center; 0.422 for aid post; 0.354 for VHV), but self-reported time varied widely among respondents with identical travel distances, reflecting differences in respondents’ life-styles and faulty recollections. Large measurement errors in variables create unreliable coefficients in empirical models, and the effect of travel time on choice of treatment provider can be underestimated [23].

Therefore, we used ordinary least squares (OLS) regression to adjust self-reported round-trip time on walking distance, transportation distance, and wait/examination time as explanatory variables (Table 2). The coefficient of wait/treatment time, however, was not significant in the regression of the health center care time and was excluded from the explanatory variables in the correction regression for the health center. As to the regression of VHVs care time, explanatory variables were walking distance and wait/treatment time; there was no one who used public transportation to visit them. The estimates of these three regressions should give us more objective value of time spent for the trip to each healthcare provider.

**Table 2:** OLS estimation of travel time spent on each healthcare visit

	Health center	Aid post	VHV
Walking distance (km)	0.198***	0.290***	0.254***
	(0.013)	(0.019)	(0.024)
Transportation distance (km)	0.133***	0.115***	
	(0.003)	(0.009)	
wait/treatment time (h)		0.605***	1.385***
		(0.101)	(0.063)
constant	3.686***	2.808***	0.363***
	(0.149)	(0.214)	(0.063)
N	1006	502	1026
R-squared	0.639	0.388	0.407

*VHV* village health volunteer

*** *p* < 0.01, ** *p* < 0.05, * *p* < 0.1

Almost all caregivers (99%) were females (i. e., mothers, grandmothers or female relatives in a household). Among females, almost all (> 99%) were non-wage workers and spent time for domestic work and farming. Therefore, difference of wage rate seemed little among the caregivers. As we did not get the information on wage rate of domestic work or agricultural work in PNG, we adopted formal minimum wage rate for estimating wage rate of study caregivers. Using these adjusted time spent for taking their children to the healthcare providers, we calculated time costs of the visits by multiplying them by the hourly minimum wage in Papua New Guinea Kina (PGK 3.2/h, PGK 1 = USD 0.38) [24].

### Descriptive statistics of variables

Table 3 shows descriptive statistics of treatment fees and transportation costs for healthcare outside the household in their explanatory variables. On average, both costs were the highest for Dagua Health Center. Moreover, the caregivers who chose the health center, paid more for transportation than for treatment, but, the caregivers who chose the aid posts, the treatment fees exceeded the transportation costs. Presumably, many caregivers, seeking for the best available care, took public transportations to the health center, but few did so to the aid posts. In contrast, all caregivers visited VHVs on foot, incurring no transportation cost.

**Table 3:** Descriptive statistics of cost-related information for health services

Cost, distance, and time	N	mean	SD	min	max
Pecuniary costs (PGK)					
Health center	455	8.48	4.73	0	23
Aid post	243	2.18	2.82	0	15
VHV	446	0.33	0.60	0	2
Treatment fee (PGK)					
Health center	462	2.76	1.31	0	10
Aid post	247	1.39	0.96	0	5
VHV	446	0.33	0.60	0	2
Transportation costs (PGK)					
Health center	455	5.73	4.51	0	20
Aid post	248	0.76	2.24	0	12
VHV	463	0	0	0	0
Time costs (PGK)					
Health center	457	26.30	7.43	12.29	39.12
Aid post	225	19.45	5.65	9.13	37.23
VHV	428	5.17	2.85	1.65	17.65
Walking distance (km)					
Health center	457	5.71	4.99	0.78	23.42
Aid post	236	7.78	5.65	0.06	23.49
VHV	475	2.03	1.77	0.00	8.33
Transportation distance (km)					
Health center	457	25.53	20.02	0.00	58.23
Aid post	236	3.79	11.55	0.00	61.23
VHV	428	0	0	0	0
Wait/treatment time (hour)					
Health center	434	1.36	1.04	0.00	8.00
Aid post	238	0.98	0.96	0.00	6.00
VHV	428	0.53	0.62	0.00	4.47

*PGK* Papua New Guinea Kina, *VHV* village health volunteer

PGK 1 = USD 0.38 (https://www.bankpng.gov.pg/historical-exchange-rates/)

As travel time using public transit is the longest to the health center for most caregivers, the average time costs are the highest for the health center, followed by the aid posts and then by the VHVs.

Also wait/treatment time was the longest at the health center. At the center, practitioners work 24 h in shifts and absenteeism is not a problem; lengthy waits were observed in the morning with the simultaneous arrival of new patients by the public transportation services. For the aid posts or for VHVs, however, our observation suggested that it was not the concentration of patients that caused lengthy waits. Within the survey area, a single health worker was assigned as a VHV or to each aid post, and many worked in their homes as farmers. Lengthy waits occurred when they had not reported for work, primarily because they were farming in their distant fields, and caregivers had to wait their return.

Time costs evaluated at minimum wage exceed pecuniary costs for all providers because public transportation and treatment fees at formal facilities are relatively inexpensive, but it takes substantial time to reach them in rural PNG.

Table 4 shows descriptive statistics for individual, caregivers, and household characteristics with respect to our explanatory variables. Average years of schooling for caregivers is 6.2 years, reflecting completion of 6 year primary school for most caregivers. Households on average have 6.7 members, and 37% keep Western pharmaceuticals (acetaminophen 24%, amoxicillin 24%, antimalarial drugs 6.5%). These drugs, generally unavailable in rural areas, presumably had been prescribed at formal health facilities or VHVs on earlier visits.

**Table 4:** Descriptive statistics of child, caregiver, and household characteristics

	N	mean	SD	min	max
Child					
Illness severity					
mild	219	(46.1)^a^			
severe	256	(53.9)^a^			
Gender					
female	227	(47.8)^a^			
male	248	(52.2)^a^			
Age (year)	475	6.023	4.056	0	14
Caregiver					
Years of schooling	475	6.242	2.868	0	13
Household					
Western medicine					
no	292	(62.1)^a^			
yes	178	(37.9)^a^			
Asset index	467	0.000	1.309	−1.182	7.117
Total number of members	472	6.689	2.684	2	19

^a^Percentage is in parentheses

Results show that 89% of households own mobile phones, 46% have access to safe drinking water, 41% own radios, 14% have metal or concrete roofs, 13% have generators, 6.4% have brick or metal walls, and 4.0% own a vehicle or outboard motorboat. The median and mode for the number of assets is 2, the average is 2.1, and 88% of households own three or fewer of the assets surveyed.

### Mixed logit model estimation

We employed a mixed logit model with home-treatment as the base alternative to estimate the choice probability among 439 observations (excluding 36 with missing values). We estimated four models; in Model 1, alternative-specific explanatory variables consist of only the pecuniary costs, in Model 2, they consist of only the time costs, in Model 3, they consist of the pecuniary costs and time costs, and in Model 4, they consist of only the total costs, or the sums of the pecuniary costs and the time costs. The coefficient of any cost in any model is significantly negative (Table 5). The random component of the pecuniary costs is 0.181 and it is statistically significant, but the random component of time costs is statistically insignificant, in other words, the effects of the pecuniary costs on provider choice vary substantially among caregivers, but the effects of time costs do not.

**Table 5:** Mixed logit model estimation

	Model 1			Model 2		
	Health center	Aid post	VHV	Health center	Aid post	VHV
Pecuniary costs	−0.269***	−0.269***	−0.269***			
	(0.079)	(0.079)	(0.079)			
Time costs				− 0.105***	− 0.105***	− 0.105***
				(0.024)	(0.024)	(0.024)
Total cost						
Illness severity	1.205***	0.775*	0.815***	1.224***	0.840**	0.875***
	(0.352)	(0.415)	(0.240)	(0.331)	(0.416)	(0.243)
Age	−0.037	−0.057	0.022	− 0.063	− 0.067	0.033
	(0.043)	(0.054)	(0.029)	(0.040)	(0.054)	(0.029)
Male	0.301	−0.593	−0.700***	0.177	−0.531	−0.759***
	(0.351)	(0.397)	(0.235)	(0.321)	(0.395)	(0.237)
Caregiver’s education	−0.026	0.107	0.035	−0.033	0.125*	0.052
	(0.064)	(0.075)	(0.042)	(0.059)	(0.075)	(0.042)
Western medicine	0.064	−0.699	0.032	0.019	−0.742*	−0.020
	(0.354)	(0.427)	(0.239)	(0.320)	(0.424)	(0.240)
Asset index	0.240*	−0.223	−0.049	0.187	−0.199	−0.066
	(0.141)	(0.195)	(0.087)	(0.118)	(0.203)	(0.088)
Household size	−0.076	− 0.188**	−0.012	−0.086	−0.169*	0.000
	(0.069)	(0.091)	(0.045)	(0.061)	(0.091)	(0.046)
Constant	0.354	0.559	−0.465	1.802**	1.801*	−0.261
	(0.812)	(0.936)	(0.519)	(0.858)	(0.986)	(0.535)
Random component						
Pecuniary costs	0.150	0.150	0.150			
	(0.061)	(0.061)	(0.061)			
Time costs				0.016	0.016	0.016
				(0.049)	(0.049)	(0.049)
Total cost						
N	439			439		
Log likelihood	− 451.463			− 446.677		
AIC	954.926			945.355		
Pseudo R2	0.082			0.091		
	Model 3			Model 4		
	Health center	Aid post	VHV	Health center	Aid post	VHV
Pecuniary costs	−0.207**	−0.207**	−0.207**			
	(0.092)	(0.092)	(0.092)			
Time costs	−0.091***	−0.091***	−0.091***			
	(0.023)	(0.023)	(0.023)			
Total cost				−0.097***	−0.097***	−0.097***
				(0.025)	(0.025)	(0.025)
Illness severity	1.375***	0.837**	0.845***	1.338***	0.851**	0.880***
	(0.372)	(0.423)	(0.242)	(0.370)	(0.430)	(0.244)
Age	0.242	−0.580	−0.755***	0.245	−0.596	−0.753***
	(0.367)	(0.404)	(0.238)	(0.356)	(0.408)	(0.239)
Male	−0.050	−0.076	0.031	− 0.061	−0.066	0.031
	(0.045)	(0.056)	(0.029)	(0.043)	(0.056)	(0.029)
Caregiver’s education	−0.057	0.130*	0.046	−0.037	0.123	0.049
	(0.068)	(0.078)	(0.042)	(0.066)	(0.078)	(0.042)
Western medicine	0.075	−0.818*	−0.033	0.023	−0.754*	−0.027
	(0.369)	(0.436)	(0.242)	(0.355)	(0.438)	(0.243)
Asset index	0.262*	−0.240	− 0.058	0.247*	−0.207	−0.063
	(0.149)	(0.206)	(0.087)	(0.139)	(0.206)	(0.088)
Household size	− 0.078	−0.176*	−0.001	−0.087	−0.179*	−0.002
	(0.072)	(0.092)	(0.046)	(0.068)	(0.093)	(0.047)
Constant	2.132**	1.984*	−0.190	1.926**	1.890*	−0.206
	(0.966)	(1.025)	(0.532)	(0.934)	(1.052)	(0.542)
Random component						
Pecuniary costs	0.181	0.181	0.181			
	(0.070)	(0.070)	(0.070)			
Time costs	0.000	0.000	0.000			
	(0.060)	(0.060)	(0.060)			
Total cost				0.033	0.033	0.033
				(0.021)	(0.021)	(0.021)
N	439			439		
Log likelihood	− 442.545			− 444.710		
AIC	941.089			941.421		
Pseudo R^2^	0.100			0.095		

*VHV* village health volunteer

*** *p* < 0.01, ** *p* < 0.05, * *p* < 0.1

**Fig. 1:**
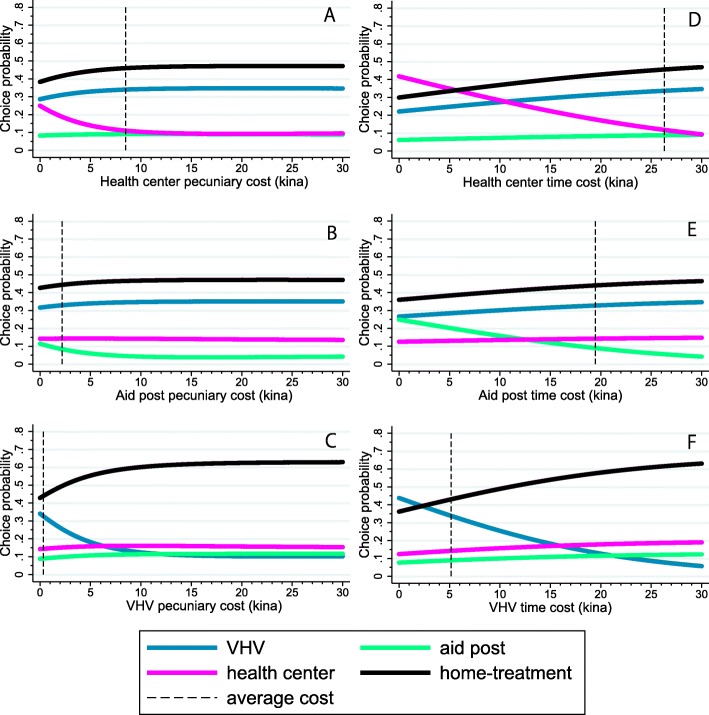
The choice probabilities of the four alternatives for a change in the pecuniary cost of the health center, aid post, or village health volunteer (left: 1**a**, 1**b**, and 1**c**) and the choice probabilities of the four alternatives for a change in the time cost of the health center, aid post, or village health volunteer (right: 1**d**, 1**e**, and 1**f**). The dashed line indicates each average cost. The unit of cost is in Papua New Guinea Kina (Kina 1 = USD 0.38). VHV: village health volunteer

Comparison of log likelihood, Akaike information criteria, and pseudo-R^2^ statistics of the four models reveals Model 3 is the best specification of the four. Consequently, we have adopted Model 3 as the basis of our counterfactual analysis regarding the pecuniary costs of a health provider without changing values of the other explanatory variables. The mean choice probability for each type of provider, given a hypothetical change in its pecuniary costs, is its average predicted choice probability. The mean choice probability curve for the alternative is downwardly convex and barely changes when the costs exceed PGK 10 (Fig. 1a–c).

With respect to the changes in own pecuniary costs, the choice probability of a VHV is the most own-price elastic, followed by Dagua Health Center and the aid posts. When the pecuniary costs of VHVs are set to 0, its choice probability is 0.34 and ranks second after home-treatment. In contrast, choice probability would not increase substantially even if pecuniary costs of the health center or the aid posts are set to zero, and the order of magnitude of their choice probability would remain unchanged. An increase in pecuniary costs for Dagua Health Center would slightly increase the choice probability of home-treatment or VHV’s, but contrary to what one might think, it would have almost no effect on the choice of the aid-posts. An increase in pecuniary costs of the aid posts exhibit similar tendencies, but the magnitude of the changes are far smaller than Health Center’s increase. An increase in pecuniary costs of VHVs would have little effect on the other two choices.

With respect to the changes in own time costs, the mean choice probability curves are close to straight lines sloping downward (Fig. 1d–f). Choice probabilities appear to be more responsive to the changes in own time costs than the changes in own pecuniary costs, comparing the slopes of the two mean choice probability curves. The probability of choosing each alternative at zero time costs exceeds that of zero pecuniary costs (0.42 vs 0.24 for Dagua Health Center, 0.25 vs 0.11 for aid posts, 0.44 vs 0.34 for VHVs). Regarding the cross-effects of time costs, for Dagua Health Center, VHVs and home-treatment are its clear substitutes, and, for the aid posts, the other three seem to be weak substitutes. On the other hand, an increase in time costs of VHVs would strongly increase the choice of home-treatment, but it would only mildly increase the choices of Health Center and the aid posts.

**Fig. 2:**
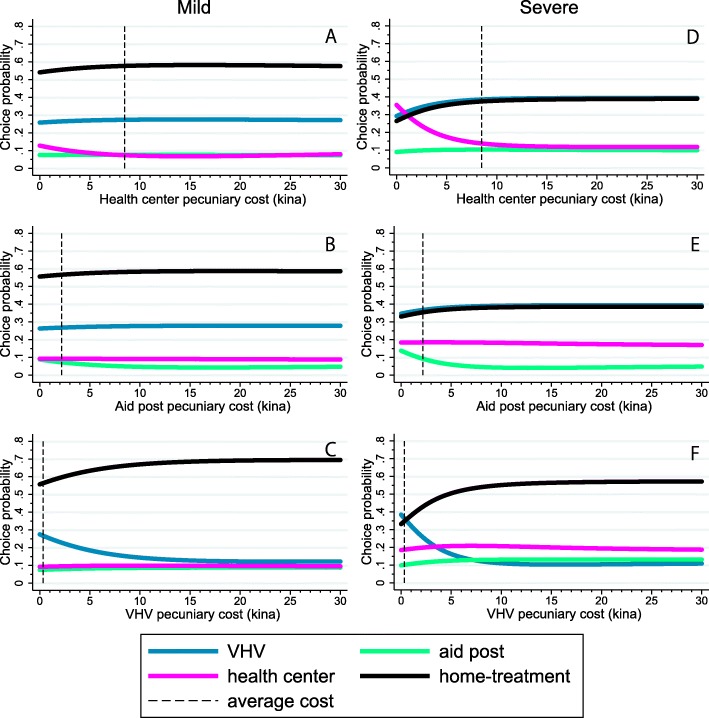
The choice probabilities of the four alternatives for a change in the pecuniary cost of the health center, aid post, or village health volunteer in the mild symptom sub-group (left: 2**a**, 2**b**, and 2**c**) and the choice probabilities of the four alternatives for a change in the pecuniary cost of the health center, aid post, or village health volunteer in the severe symptom sub-group (right: 2**d**, 2**e**, and 2**f**). The dashed line indicates each average cost. The unit of cost is in Papua New Guinea Kina (Kina 1 = USD 0.38). VHV: village health volunteer

Among individual-specific variables, severity of illness increases the probability of choice for each type of healthcare providers over the home-treatment (Table 5). Therefore, we performed a sub-group analysis for severity. Given mild symptoms, pecuniary costs exerted relatively little effects on the probability of choosing the providers, but, for children with severe symptoms, they were much more significant (Table 6). For example, when the pecuniary costs are set to zero, caregivers of children with mild symptoms would not much increase the choice of healthcare services (Fig. 2a–c), but more caregivers of children with severe symptoms would choose either the health center or VHVs than home-treatment (Fig. 2d–f).

**Table 6:** Illness severity sub-group analysis

	Mild sub-group			Severe sub-group		
	Health center	Aid post	VHV	Health center	Aid post	VHV
Pecuniary costs	− 0.125	−0.125	−0.125	−0.303**	−0.303**	−0.303**
	(0.134)	(0.134)	(0.134)	(0.145)	(0.145)	(0.145)
Time costs	−0.122***	−0.122***	−0.122***	−0.086***	−0.086***	−0.086***
	(0.039)	(0.039)	(0.039)	(0.032)	(0.032)	(0.032)
Age	−0.058	0.039	−0.008	−0.030	−0.177**	0.066
	(0.064)	(0.086)	(0.043)	(0.072)	(0.085)	(0.042)
Male	0.041	−0.906	−0.665*	0.516	−0.596	−0.851**
	(0.535)	(0.656)	(0.356)	(0.574)	(0.569)	(0.332)
Caregiver’s education	0.120	0.178	0.075	−0.158	0.079	0.023
	(0.117)	(0.142)	(0.067)	(0.099)	(0.106)	(0.055)
Western medicine	−0.070	−0.387	−0.010	0.082	−1.216**	−0.056
	(0.549)	(0.705)	(0.364)	(0.561)	(0.599)	(0.337)
Asset index	0.310	0.065	−0.102	0.308	−0.549*	−0.062
	(0.211)	(0.260)	(0.172)	(0.246)	(0.319)	(0.106)
Household size	0.039	−0.175	−0.014	−0.159	−0.163	0.003
	(0.110)	(0.163)	(0.083)	(0.108)	(0.117)	(0.057)
Constant	0.707	1.509	−0.003	4.443***	3.662***	0.609
	(1.561)	(1.664)	(0.806)	(1.418)	(1.398)	(0.677)
Random component						
Pecuniary	0.128	0.128	0.128	0.273	0.273	0.273
	(0.092)	(0.092)	(0.092)	(0.125)	(0.125)	(0.125)
Time costs	0.002	0.002	0.002			
	(0.066)	(0.066)	(0.066)			
N	198			241		
Log likelihood	−182.946			− 250.154		
AIC	415.892			548.308		
Pseudo R^2^	0.099			0.110		

*VHV* village health volunteer

In the severe sub-group, a random component for time costs was not included because the model did not converge when random components were assumed for both pecuniary and time costs

***p < 0.01, **p < 0.05, *p < 0.1

**Fig. 3:**
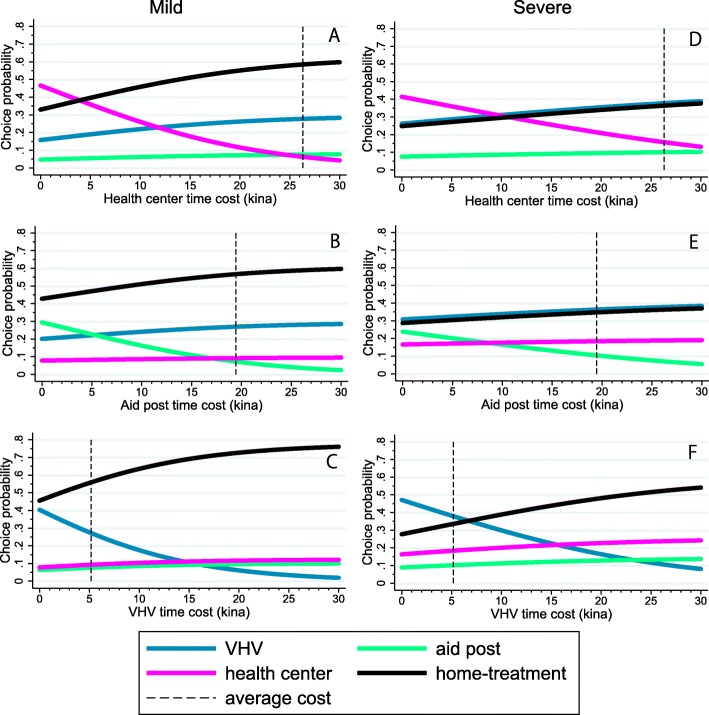
The choice probabilities of the four alternatives for a change in the time cost of the health center, aid post, or village health volunteer in the mild symptom sub-group (left: 3**a**, 3**b**, and 3**c**) and the choice probabilities of the four alternatives for a change in the time cost of the health center, aid post, or village health volunteer in the severe symptom sub-group (right: 3**d**, 3**e**, and 3**f**). The dashed line indicates each average cost. The unit of cost is in Papua New Guinea Kina (Kina 1 = USD 0.38). VHV: village health volunteer

Irrespective of severity of symptoms, increases in time costs significantly reduce the probability of choosing all providers (Table 6). For children with severe symptoms (Fig. 3d–f), however, the choice probability diminishes more slowly than for children with mild symptoms as time costs increases (Fig. 3a–c).

The probability of choosing VHVs is significantly higher when the patient child is female. At 10% significance, holding of Western medicine, a higher-educated caregiver, and small household size increase the probability of choosing an aid post. Larger assets raised the probability of choosing a health center (Table 5). In sub-group analysis, these effects are significant only for severe symptoms (Table 6).

## Discussion

In selecting healthcare providers for febrile children, we have found that their caregivers respond more to changes in the time costs than changes in the pecuniary costs. Counterfactual analysis reveals that the probability of choosing a provider with zero time costs is higher than choosing an identical one with zero pecuniary costs. Therefore, reducing time costs rather than pecuniary costs should be more effective in promoting the use of health services. Furthermore, according to our mixed logit estimation results, policies targeting to reduce time costs likely have higher internal validity than policies targeting to reduce pecuniary costs. This is because the effects of time costs on provider choice are similar among caregivers, while the effects of pecuniary costs among caregivers can vary substantially among caregivers due to their prominent individual heterogeneity. These findings commend a policy of reducing the time costs of health services.

From the view-point of VHVs time costs, home-treatment is far-closer substitute than the other two healthcare facilities. Reducing the time costs of VHVs would have minor negative impacts on the choice of these facilities, but would induce a substantial behavioral change from home-treatment to VHVs. Reducing the time costs of VHVs should raise total demand for health services. In many cases, patients are kept waiting because VHVs are farming in their own fields instead of waiting for patients who may come. Unsalaried VHVs have little incentive to serve the residents of their community, but paying them at least the minimum wage may provide them the incentive.

Policies that reduce travel time to formal health facilities may include appropriate geographical relocation, increased public transportation, and road maintenance. Expenses of these policies could be prohibitive in LMIC. Even for them, however, policies to reduce wait times at these facilities can be implemented at small costs. Appointments by email, short-message services, and websites improve access and reduce wait times [25, 26]. Even in LMIC, a stated preference study endorses a short-message service-based appointment system [27]. Given that about 90% of residents in our surveyed area own mobile phones, health facilities may be able to reduce wait times by scheduling appointments using inexpensive text messaging.

In addition, rates of follow-up visits likely can be increased by using the reservation system at each health facility and better outcome can be expected. Exchanging information by text message may establish intra-system cooperation, such as referrals from VHVs to aid posts and health centers and reverse referrals from health centers to VHVs and aid posts [28].

Wait times at Dagua Health Center rose because of patient congestion in the morning and waiting for public transportation to return home. Introducing the scheduled appointment system described above could reduce the former. Reducing time of patients awaiting public transport may be possible by using one of the two ambulances at Dagua Health Center. Cost effectiveness analysis could clarify the added expenses of this policy.

Aid post workers also are sometimes absent during working hours. They receive fixed salaries regardless of patient load, but docking their salaries for absences may not be easy. The more important issue is that in remote communities, they are not substitutes for the health center, because many potential patients are unaware of them. Further studies are needed to investigate whether such unawareness is attributable to supply-side factors such as inadequate quality of healthcare services or demand-side factors such as community characteristics.

Selecting and qualifying skilled VHVs as aid post workers after completing training may strengthen the function of the aid posts. Although that entails substantial expenditure for training, construction, and operation of facilities, health workers accepted by their villages should have high retention rates and become key providers of rural healthcare. Opportunity for promotion could motivate current VHVs.

Although pecuniary costs of health services is less elastic than time costs, raising treatment fees, especially for VHVs, should not be easily carried out. Demand for their services in the area studied is not excessive, even when treatment is free. If pecuniary costs for services were nearly zero, small out-of-pocket payments could reduce the probability of choosing their provider, although patients who need treatment might go elsewhere.

The probability of choosing a healthcare provider is more elastic with respect to changes in pecuniary costs. Overall, severity of symptoms little affects it with regard to time costs, but the response to pecuniary costs is heterogeneous among caregivers: some visited a provider regardless of pecuniary costs, whereas others reacted strongly to changes in pecuniary costs. That was more prominent with severe symptoms. Therefore, it is necessary for the severely ill not to avoid health-service visits.

This study has several limitations. First, recall bias may have occurred because our questionnaire asks caregivers to recall events of the two previous weeks. The actual occurrence of fevers may have been higher and visits to healthcare providers fewer because caregivers did not recall minor symptoms or regarded illnesses as asymptomatic. Second, our model did not measure VHVs’ clinical skills, potentially a factor affecting patients’ choice of healthcare provider. Its exclusion might foster estimation bias. Third, we studied only one administrative area of PNG. Studies elsewhere are needed to provide external validity.

## Conclusions

Using a mixed logit model, we investigated how pecuniary costs or time costs of healthcare providers affect caregiver’s choice of treatment for his/her febrile child in rural PNG. Further, we altered pecuniary costs and time costs for each healthcare provider to compare probability of choosing a type of healthcare provider counterfactually. The features of our model are that we included pecuniary costs (including transportation cost) and time costs as explanatory variables, and we estimated our model using stated cost information, resulting in that a treatment choice set with individual heterogeneity (the probability of choosing unrecognized health services was set to 0). Compared to reducing pecuniary costs, reducing time costs significantly affected the probability that caregivers would choose treatment by VHVs, an aid post, or a health center. We also observed individual heterogeneity in effects of pecuniary costs on choice of healthcare provider. Although IMIC have restricted health and financial resources, they need to develop policies to reduce time costs and pecuniary costs to improve access to essential healthcare.

## Data Availability

The datasets used and/or analyzed in the current study are available to the extent permitted by Papua New Guinea Department of Health and the Tokyo Women’s Medical University policies.

## Abbreviations

GPS: global positioning system
LMIC: low and middle income countries
OLS: ordinary least square
PMV: Public Motor Vehicle
PNG: Papua New Guinea
UHC: universal health coverage
VHV: village health volunteer
